# Targeted delivery and stimulus-responsive release of anticancer drugs for efficient chemotherapy

**DOI:** 10.1080/10717544.2021.1986602

**Published:** 2021-10-20

**Authors:** Lei Qiao, Xue Yuan, Hui Peng, Guisong Shan, Min Gao, Xiaoqing Yi, Xiaoyan He

**Affiliations:** a School of Basic Medical Sciences, Anhui Medical University, Hefei, China; b Inflammation and Immune Mediated Diseases Laboratory of Anhui Province, School of Life Sciences, Anhui Medical University, Hefei, China; c Department of Respiratory and Critical Care Medicine, The First Affiliated Hospital of Anhui Medical University, Hefei, China; d Key Laboratory of Prevention and Treatment of Cardiovascular and Cerebrovascular Diseases, Ministry of Education, Gannan Medical University, Ganzhou, China

**Keywords:** Chemotherapy, CaCO_3_, targeted delivery, acid-response, mitochondria, nucleus

## Abstract

Chemotherapy is currently an irreplaceable strategy for cancer treatment. Doxorubicin hydrochloride (DOX) is a clinical first-line drug for cancer chemotherapy. While its efficacy for cancer treatment is greatly compromised due to invalid enrichment or serious side effects. To increase the content of intracellular targets and boost the antitumor effect of DOX, a novel biotinylated hyaluronic acid-guided dual-functionalized CaCO_3_-based drug delivery system (DOX@BHNP) with target specificity and acid-triggered drug-releasing capability was synthesized. The ability of the drug delivery system on enriching DOX in mitochondria and nucleus, which further cause significant tumor inhibition, were investigated to provide a more comprehensive understanding of this CaCO_3_-based drug delivery system. After targeted endocytosis by tumor cells, DOX could release faster in the weakly acidic lysosome, and further enrich in mitochondria and nucleus, which cause mitochondrial destruction and nuclear DNA leakage, and result in cell cycle arrest and cell apoptosis. Virtually, an effective tumor inhibition was observed *in vitro* and *in vivo*. More importantly, the batch-to-batch variation of DOX loading level in the DOX@BHNP system is negligible, and no obvious histological changes in the main organs were observed, indicating the promising application of this functionalized drug delivery system in cancer treatment.

## 1. Introduction

Although considerable progress has been achieved in the fields of radiotherapy and surgery, chemotherapy still plays an irreplaceable role in the treatment of various types of cancer (Shi et al., 2017; Wu et al., 2017; Wang et al., 2020; Zhang et al., 2020). Doxorubicin hydrochloride (DOX) is one of the most commonly used antibiotics that can effectively reduce the growth and progression of cancer through several pathways, such as mitochondrial depolarization and nuclear DNA damage (Song et al., 2015; Xiong et al., 2016). However, the main hurdles that limit its use include severe toxicity, low bioavailability, serious adverse effects on normal tissues, and even cancer relapse in many clinical indications (Perez-Herrero & Fernandez-Medarde, 2015; Kim et al., 2020; Mi et al., 2020).

Nanoparticles that can selectively and efficiently deliver drugs to tumor tissues and kill malignant cells have attracted great research interest as one of the most promising strategies to address these issues (Torchilin, 2011; Cabral & Kataoka, 2014; Sindhwani et al., 2020; Izci et al., 2021). In the past decade, a variety of vectors, such as cationic liposomes (Vaidya et al., 2019), polymer micelles (Yang et al., 2020), cell exosomes (Barile & Vassalli, 2017), and gold/magnetic nanoparticles (Singh et al., 2018) with targeting ligands (e.g. proteins, small molecules, and polysaccharides) have been employed to construct drug delivery carriers to facilitate tumor accumulation and induce tumor cell death (Zhang et al., 2018; Yoo et al., 2019). However, there are some limitations to the clinical use of nanoparticle-mediated DOX therapy, in particular, the complex preparation process, which may lead to batch-to-batch variation of DOX loads. More importantly, hampered DOX release and inefficient intracellular target enrichment (e.g. mitochondria and nucleus) after nanoparticles administration may also hinder it therapeutic efficacy.

Hitherto, diverse drug release mechanisms have attracted the interest of researchers to address this issue. They take advantage of the environmental differences between tumor cells and normal cells, such as lower oxygen levels and pH, higher thiol levels, and enzyme overexpression, to achieve the controlled release of anticancer drugs while reducing the side effects to normal tissues (Shi et al., 2020; Ma et al., 2021; Yi et al., 2021; Zha et al., 2021). In addition, to the best of our knowledge, calcium carbonate (CaCO_3_) is sensitive to pH changes and can achieve controlled drug release through the acidic extracellular environment in solid tumor tissues and in the lysosomes inside cancer cells. Furthermore, Ca^2+^ and CO_3_^2−^ are naturally occurring ions, inherently possessing good biocompatibility and biosafety. Its capability to load various drugs and its large specific surface area also makes CaCO_3_ an ideal drug vector for use in the treatment of cancer (Zhao et al., 2014; Dong et al., 2016; Dizaj et al., 2019; Xu et al., 2020). As it is well known, mitochondria and nucleus are two important intracellular therapeutic targets by DOX (Song et al., 2015; Xiong et al., 2016). Previous studies on DOX-loaded CaCO_3_ delivery system (DOX/CaCO_3_ system) are mainly focused on the effects of system on cell growth inhibition, cell cycle arrest, and cell apoptosis (Peng et al., 2017; Li et al., 2020; Yang et al., 2021). As far as we know, inadequate attention has been paid to study the effect of DOX/CaCO_3_ system on its intracellular therapeutic targets. The purpose of our study is to develop a DOX/CaCO_3_ system to effectively inhibit tumor growth as well as to study the effects of the system on mitochondrial morphologies, nuclear DNA damage, and diverse proteins involved in these processes to provide a more comprehensive understanding in this field.

**Scheme 1.**
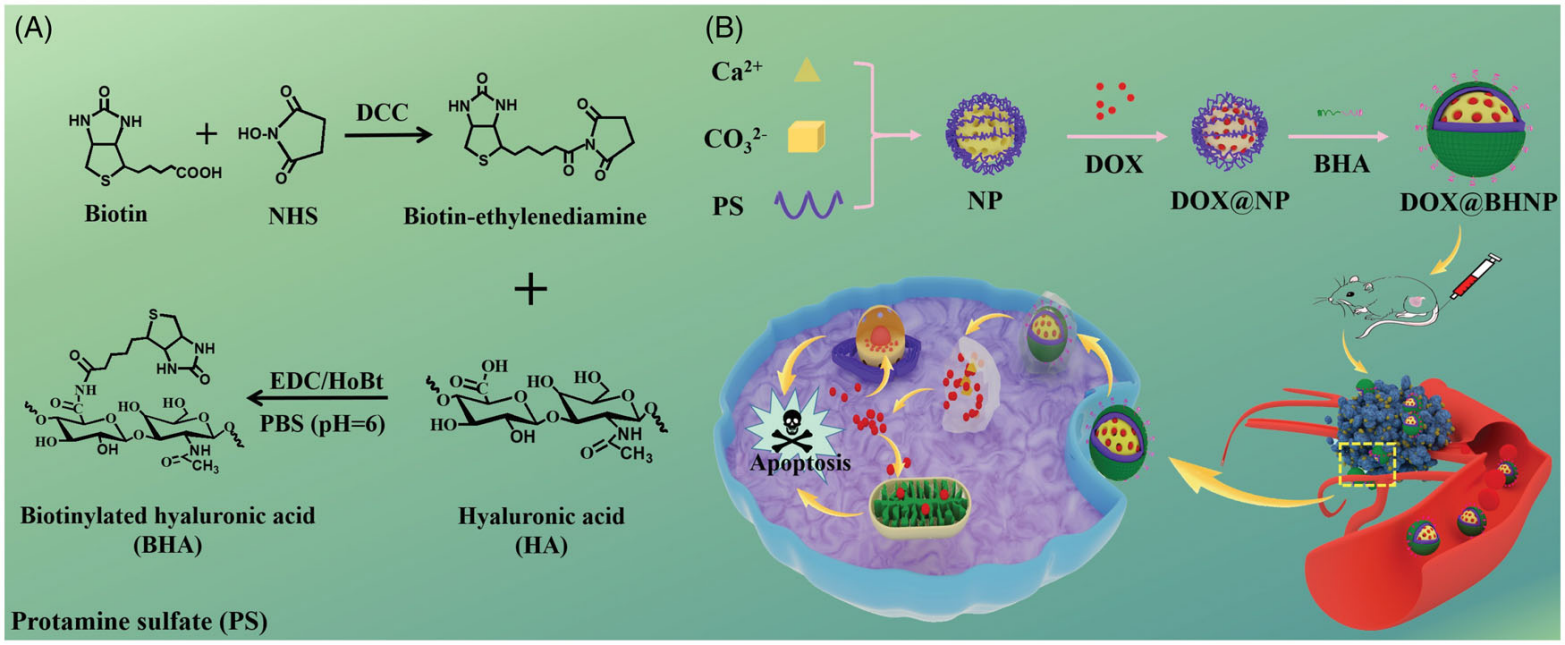
(A) Procedures for preparing the biotinylated hyaluronic acid (BHA). (B) Schematic diagram of the synthetic procedures for DOX@BHNP and the intracellular acid-triggered controlled drug release for cancer treatment.

Herein, we used a modified nanoprecipitation technique to prepare a biotinylated hyaluronic acid (BHA)-guided pH-responsive CaCO_3_ nanoparticle system to achieve targeted and controlled DOX delivery and provide effective cancer therapy (Scheme 1). As depicted in Scheme 1, the DOX is encapsulated in the core composed of protamine sulfate (PS) and CaCO_3_, then BHA is modified onto the surface of the core. Biotin (vitamin B7) has been reported to bind to biotin receptors that are overexpressed on the surface of various tumor cells (Segura et al., 2005; Ren et al., 2015; Jelonek et al., 2019). Negatively charged hyaluronic acid (HA) has good biocompatibility, is capable of self-assembly, and improves tumor cell targeting efficiency because of the high expression of CD44 in various tumor cells (Mattheolabakis et al., 2015; He et al., 2018; Huang & Huang, 2018). With the aid of these functional components, the delivery system we fabricated can specifically deliver the anticancer drug DOX to tumor cells and mediate its efficient diffusion to the mitochondria and nuclei of its target cells.

To confirm the functions of each component in the delivery vector, the physicochemical properties, antitumor efficacy, and systemic effects of the DOX-loaded nanoparticles were assessed. Briefly, because of the high specificity and affinity of BHA to tumor cells, the most obvious enrichment of DOX was observed in the tumor cells treated with DOX@BHNP. Due to the pH sensitivity of CaCO_3_, the nanoparticles were disassembled in the endosome and lysosome after endocytosis, and an obvious enrichment of DOX in the mitochondria and nuclei of the target cells was observed in the *in vitro* study. Consequently, DOX@BHNP system significantly destroyed the mitochondria, induced DNA damage, regulated the expression of diverse proteins involved in these process, caused cell cycle arrest and tumor cell apoptosis. The enhanced tumor inhibitory effects and reduced side effects observed in both *in vitro* and *in vivo* studies indicate the promising application of dual-functionalized nanoparticles in cancer treatment.

## 2. Materials and methods

### 2.1. Materials

Hyaluronic acid was purchased from Shandong Freda Biochem Co., Ltd. (Jinan, China). Protamine sulfate, biotin, and ethylenediamine were purchased from Sigma-Aldrich (Shanghai, China). Analytical grade anhydrous calcium chloride (CaCl_2_) and anhydrous sodium carbonate (Na_2_CO_3_) were obtained from ANPEL Laboratory Technologies Co., Ltd. (Shanghai, China). 3-(4,5-Dimethylthiazol-2-yl)-2,5-diphenyltetrazolium bromide (MTT) was supplied by Amresco (Dallas, TX). Dimethylsulfoxide (DMSO) was purchased from Sigma-Aldrich (Shanghai, China). Doxorubicin hydrochloride, dimethylformamide (DMF), dicyclohexylcarbodiimide (DCC), N-hydroxysulfosuccinimide (sulfo-NHS), 1-ethyl-3-(dimethylaminopropyl)-carbodiimide (EDC), and 1-hydroxybenzotriazole (HOBt) were provided by J&K Scientific Co., Ltd. (Shanghai, China). 5,5′,6,6′-Tetrachloro-1,1′,3,3′-tetraethylbenzimidazolyl-carbocyanine iodide (JC-1) was purchased from Beyotime Biotechnology (Shanghai, China). The antibodies of p-H_2_AX, GAPDH, Bcl-2, and cleaved caspase-3 were provided by CST. Detailed information on the suppliers of cells and animals are provided in the Supporting Information (SI).

### 2.2. Synthesis of biotinylated hyaluronic acid

Biotin (2.44 g), DCC (2.47 g), and NHS (1.38 g) were added to a solution of DMF (10 mL) under continuous stirring and were reacted at 30 °C for 2 h. Then, the solvent was evaporated and precipitated in acetone to obtain biotin–NHS. Ethylenediamine trimethylamine (5 mL) was then added and the mixture was stirred at 30 °C for another 12 h to obtain biotin-ethylenediamine. After that, 170 mg of HA was dispersed in PBS buffer (1 mL, pH = 6.0) and activated, and 70 mg of biotin-ethylenediamine was added and continuously stirred at 30 °C for 24 h. The resulting solution was dialyzed and lyophilized to obtain BHA. Specific details about this experiment are provided in the SI.

### 2.3. Construction and characterization of drug-loaded nanoparticles

Deionized water was used to dissolve the materials and their compositions were as follows: component A (35 μL): 30 μL Ca^2+^ solution (CaCl_2_, 0.02 M)+5 μL anticancer DOX solution (10 μg µL^−1^); component B (55 μL): 40 µL CO_3_^2–^ solution (Na_2_CO_3_, 0.01 M) and 15 µL PS solution (2 μg µL^−1^). Subsequently, component B was added to component A dropwise and mixed gently for 10 min to obtain a solution (90 μL) containing the hybrid nanoparticles DOX@PS/CaCO_3_ (hereafter termed as ‘DOX@NP’). Mono-functionalized drug-loaded nanoparticles DOX@HA/PS/CaCO_3_ (hereafter termed as ‘DOX@HNP’) were prepared by gently mixing 10 µL of HA solution (1 μg µL^−1^) with the DOX@NP solution for 10 min. Dual-functionalized DOX-loaded nanoparticles DOX@BHA/PS/CaCO_3_ (hereafter termed as ‘DOX@BHNP’) were obtained by gently mixing the solution containing BHA (10 µg) with the DOX@NP solution (90 µL) for 10 min. The drug-loading capacity (DLC) and drug encapsulation efficiency (DEE) of the nanoparticles were determined through their absorbance at 488 nm using a spectrophotometer. Using an external calibration standard curve, the fluorescence intensity was converted into the concentration of non-encapsulated DOX, obtaining the DLC and DEE. A Zetasizer (Malvern Zetasizer Nano ZS90, Malvern, UK) was used to measure the size and zeta potential of the nanoparticles suspended in deionized water. All the data in our study were reported based on three independent measurements. The morphology of the BHA-functionalized drug delivery system was imaged using transmission electron microscopy (TEM) (Talo L120C G2).

### 2.4. *In vitro* drug release assay

The drug release properties of DOX from our synthesized delivery system were investigated (DOX amount: 200 µg) using a dialysis bag (MWCO 3500) containing 4 mL of a drug-loaded nanoparticle suspension immersed in 10 mL of a Tris–HCl solution (pH = 5.3, 6.5, and 7.4). The samples were continuously oscillated in a shaking water bath at a speed of 150 rpm at a constant temperature of 37 °C. One milliliter of the sample was taken from the solution at different time points for fluorescence spectroscopy, and then replaced with the same volume of fresh medium. The concentration of released DOX was measured using a Cary Eclipse fluorescence spectrophotometer (Agilent Technologies, Santa Clara, CA). The fluorescence intensity of the blank Tris–HCl solution (pH = 5.3, 6.5, and 7.4) was also measured and used for correction. The experimental procedure performed was as previously described by Xu et al. (2019).

### 2.5. *In vitro* cell uptake assay

Cell internalization was qualitatively observed using a confocal laser scanning microscope (CLSM) (Zeiss, LSM800, Oberkochen, Germany). Briefly, 1 × 10^5^ cells were seeded into special petri dishes and stabilized before incubation with different reagents at a DOX concentration of 6 μg mL^−1^ for 4 h and then carefully washed with PBS. Additionally, HO-(CH_2_O)*_n_*-H was added to fix the cells which were cultured in an 35 °C incubator for 15 min. To avoid interference, the fixed cells were rinsed again with PBS after incubation. Hoechst 33342 was used to stain the cells nuclei for 15 min which were then washed with PBS. The fluorescence of Hoechst 33342 and DOX was then directly determined via CLSM. Cellular uptake was quantitatively studied using flow cytometry (CytoFLEX S). Briefly, the cells in the culture medium were seeded in a six-well plate at a density of 2 × 10^5^ cells per well. After culturing for 24 h, the culture medium was replaced with 2 mL of fresh medium containing a particular reagent with a DOX concentration of 6 μg mL^−1^. After 4 h of co-incubation, the cells were washed three times with PBS, dislodged using a trypsin solution, centrifuged at 1500 rpm at 4 °C for 3 min, and then re-suspended in PBS. Finally, the cells were filtered and analyzed via flow cytometry.

### 2.6. *In vitro* cytotoxicity assay

MTT was used to evaluate the cytotoxicity of the different drug-loaded nanoparticles (Hao et al., 2021). Briefly, after treating the cells with blank nanoparticles, free DOX, DOX@NP, DOX@HNP, and DOX@BHNP at different DOX concentrations, 20 µL MTT solution was added, and the resulting formazan product was dissolved in DMSO solution. The optical density (OD) value at 570 nm was measured using a microplate reader (Bio-Rad 550, Hercules, CA). The OD value of the cells incubated in culture medium containing 10% fetal bovine serum (FBS) was also measured as a control. In addition, after the tumor cells were incubated with DOX@BHNP for 4 h, mitochondrial morphology was observed via bio-TEM. Specific details about this experiment are provided in the SI.

### 2.7. Cell apoptosis assay

Cell apoptosis was quantitatively studied using flow cytometry. Briefly, cells suspended in the culture medium were seeded in a six-well plate at a density of 2 × 10^5^ cells per well. After incubation with free DOX, DOX@NP, DOX@HNP, and DOX@BHNP at a DOX concentration of 6 µg mL^−1^, the tumor cells were collected and resuspended in PBS. Apoptotic cells were detected using an Annexin V-Pacific Blue/SYTOX Double Stain Apoptosis Detection Kit via flow cytometry. For comparison, cells incubated in a culture medium containing 10% FBS were used as a control.

### 2.8. Cell cycle assay

In this assay, the cells were quantitatively analyzed using flow cytometry. Briefly, cells were seeded in a six-well plate at a density of 2 × 10^5^ cells per well. After incubation with DOX@BHNP at a DOX concentration of 6 µg mL^−1^, the cells were harvested, washed with PBS, and fixed in chilled 75% ethanol for 24 h at 4 °C. The cells were then treated with a mixture of RNase A and SYTOX for 30 min at 37 °C. The cell cycle distribution was examined using flow cytometry. The DNA content at different phases of the cell cycle was also analyzed using ModFit software (Verity Software House, Topsham, ME).

### 2.9. Mitochondrial membrane potential determination

The mitochondrial membrane potential of 4T1 cells was determined via staining with 5,5′,6,6′-tetrachloro-1,1′,3,3′-tetraethylbenzimidazolylcarbocyanine iodide (JC-1) and detected using a fluorescence spectrophotometer. Specific details about this experiment are provided in the SI.

### 2.10. Western blot assay

Briefly, 4T1 cells were seeded in a six-well plate at a density of 2 × 10^5^ cells per well. After co-incubated with fresh culture medium containing a particular agent (free DOX, DOX@NP, DOX@HNP, and DOX@BHNP, respectively) for 24 h, the cells were rinsed twice with precooled PBS and lysed to extract proteins for western blot assay. The western blot assay processes are described in detail in the SI.

### 2.11. *In vivo* chemotherapy

When their tumor volumes reached approximately 80 mm^3^, 4T1 tumor-bearing mice were randomly divided into three groups and administered an intravenous injection of either (i) PBS, (ii) free DOX, or (iii) DOX@BHNP group, at a dosage of 6 mg kg^−1^ DOX in 100 μL PBS every two days for up to 12 days. The body weight of the mice and their tumor volumes were recorded during the experimental period, before euthanization, and during imaging. After treatment for 18 days, the tumors, hearts, livers, spleens, lungs, and kidneys of the mice were collected, sectioned, and stained with hematoxylin and eosin (H&E). In addition, the tumors were harvested for western blot and immunohistochemical (IHC) assay, Ki67 and the TUNEL staining. The details are provided in the SI.

## 3. Results and discussion

### 3.1. Preparation and characterization of the nanoparticles

Herein, an anti-cancer drug delivery system was prepared using a classic nano-precipitation technique, where it was functionalized to specifically target tumor ligands using BHA. The schematic synthesis of BHA is described in Scheme 1 and Figure S1A, where BHA was also characterized via ^1^H NMR spectroscopy (Bruker AM 400, Rheinstetten, Germany). As shown in Figure S1B, the characteristic peaks between 2 and 3 of biotin were observed in BHA (within the red dotted line), indicating its successful synthesis.

**Figure 1.**
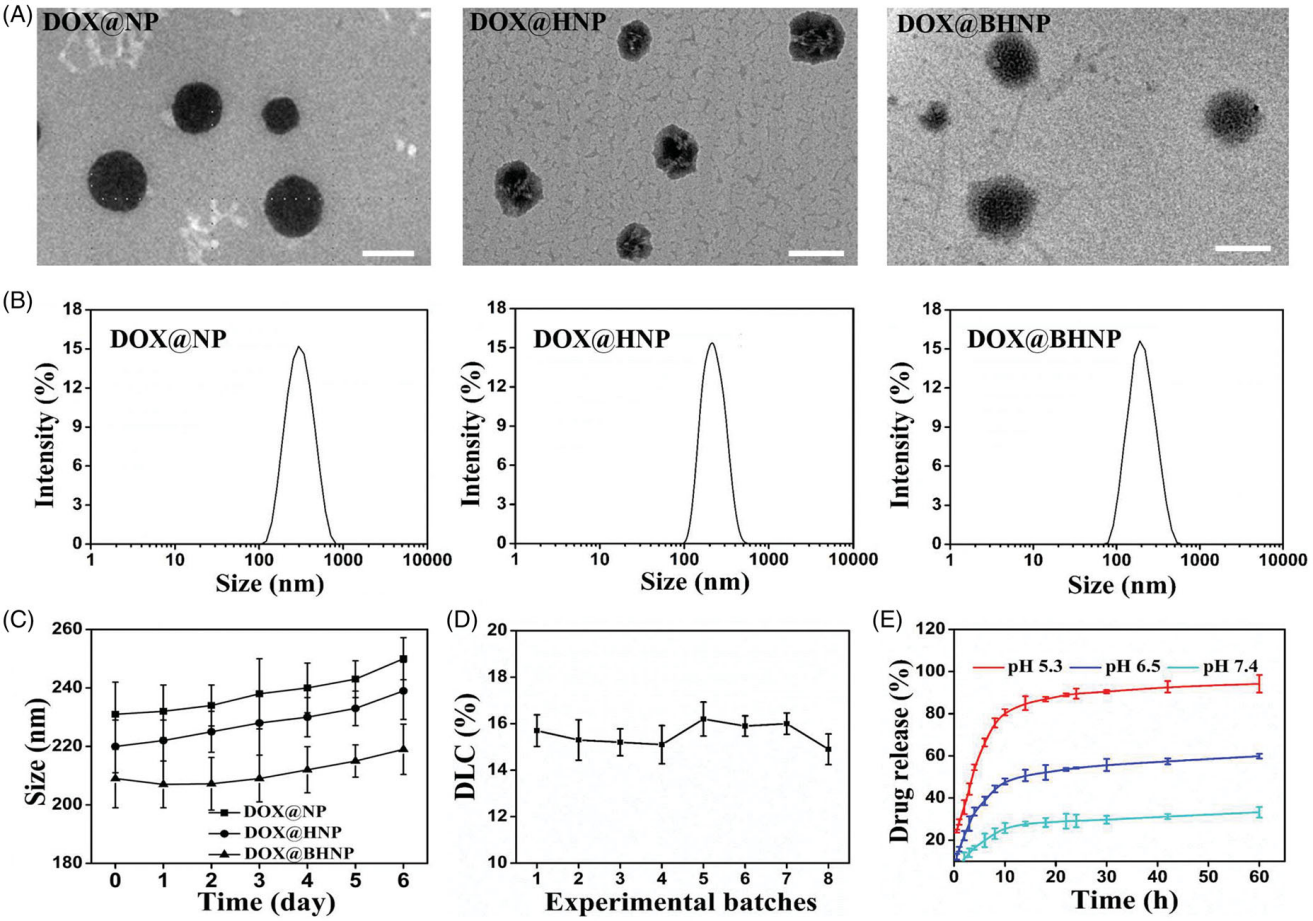
(A) TEM images of DOX@NP, DOX@HNP, and DOX@BHNP nanoparticles (scale bar: 200 nm). (B) Size distribution and (C) size change during the storage of different DOX-loaded nanoparticles as determined via DLS. (D) DLC (%) of DOX loaded in DOX@BHNP nanoparticles at different experimental batches. (E) *In vitro* release of DOX from the DOX@BHNP system in Tris–HCl solutions with different pH (5.3, 6.5, and 7.4). Error bars indicate S.D. (*n* = 3).

Furthermore, to prepare the dual-functionalized drug delivery system, negatively charged BHA was introduced into positively charged co-precipitated DOX@PS/CaCO_3_ cores to form DOX@BHA/PS/CaCO_3_ nanoparticles (DOX@BHNP) via self-assembly through electrostatic forces (Scheme 1). In this system, CaCO_3_ is a well-known acid stimulus-responsive drug delivery vehicle that can achieve effective drug release in tumor cell lysosomes. Additionally, PS as a template macromolecule could provide binding sites for Ca^2+^ and promote the formation of well-dispersed CaCO_3_ nanoparticles with excellent cell-penetrating properties and nucleus-targeting abilities. At the same time, its positive characteristics are conducive to the assembly of negatively charged functional ligands such as BHA to the delivery system. BHA endows the nanoparticles with tumor-targeting ability via an interaction with CD44 and biotin receptors on the tumor cells, as well as improve the stability of drug-loaded nanoparticles in the blood circulation. The entrapment efficiency and drug-loading data are presented in Table 1. All tested NPs showed a narrow size distribution and the entrapment efficiency and DLC of DOX in the NPs were more than 85% and 11.6%, respectively, implying that DOX was encapsulated into the network complex of PS and CaCO_3_ effectively. More importantly, the batch-to-batch variation of DOX loading level in DOX@BHNP system was studied, and it was proved that the variation of the synthetic download amount of different batches was negligible (Figure 1(D)).

**Table 1.** Size, PDI, zeta potential, DLC (%), and DEE (%) of DOX loaded in different materials.

Sample	Size (nm)	PDI	Zeta potential (mV)	DLC (wt %)	DEE (%)
DOX@CaCO_3_	453 ± 38	0.47	–5.2 ± 1.0	7.3	70.3
DOX@PS/CaCO_3_	231 ± 11	0.21	18.3 ± 1.2	11.6	85.5
DOX@HA/PS/CaCO_3_	220 ± 9	0.20	–14.2 ± 0.9	13.8	86.4
DOX@BHA/PS/CaCO_3_	209 ± 10	0.16	–13.7 ± 0.8	15.4	89.7

To evaluate the effect of BHA on the physical and chemical properties of the dual-functionalized nanoparticles, nanoparticles with different BHA content ranging from 5 to 20 µg were prepared and analyzed via DLS and flow cytometry. As shown in Figure S2, when the amount of BHA was 10 µg, the mean fluorescence intensity (MFI) of the cells was relatively higher. Thus, in this study, we fixed the amount of BHA to 10 µg to prepare the DOX@BHNP nanoparticles (Duan & Li, 2013). The TEM images of DOX@NP, DOX@HNP, and DOX@BHNP particles (Figure 1(A)) respectively display a regular spherical morphology, and are homogeneously dispersed as individual particles in distilled water, with an average diameter of approximately 200 nm, similar to the size measured via DLS (Figure 1(B)). Owing to the adsorption of HA and BHA, the surface of DOX@HNP and DOX@BHNP is covered with a transparent film (Figure 1(A)). Furthermore, the stability of a drug delivery system is a crucial factor for its bioavailability. Hence, the morphologies of particles on the 6th day were investigated and found that the increase in particle size and the change in morphology were negligible (Figure 1(C), Figures S3–S4).

**Figure 2.**
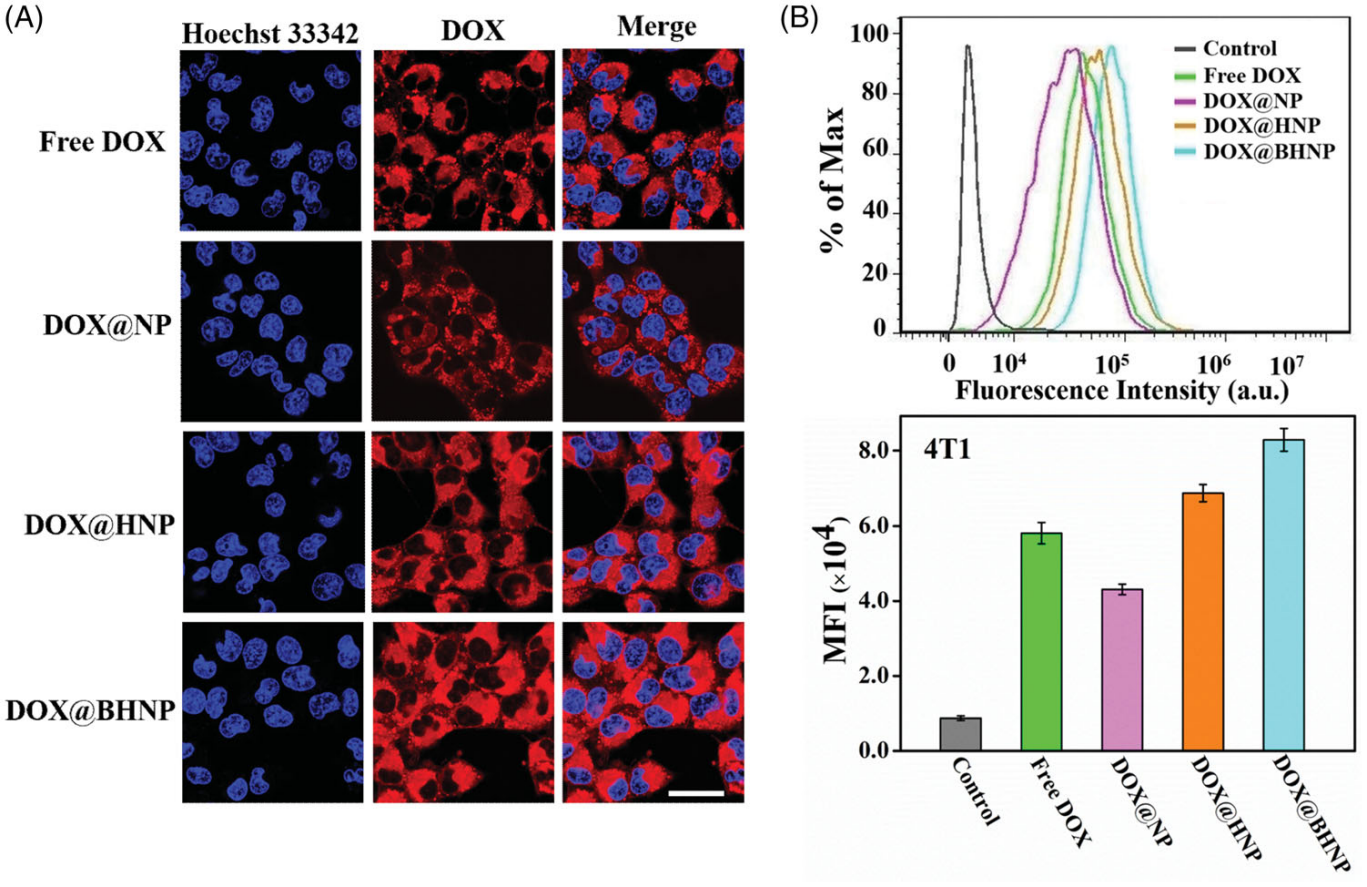
(A) CLSM images and (B) flow cytometry analysis of the intracellular DOX levels in 4T1 cells incubated with different agents (free DOX, DOX@NP, DOX@HNP, and DOX@BHNP at a DOX concentrations of 6 µg mL^−1^) for 4 h. The cells without treatment were used as control. Scale bar: 30 µm. Error bars indicate S.D. (*n* = 3).

**Figure 3.**
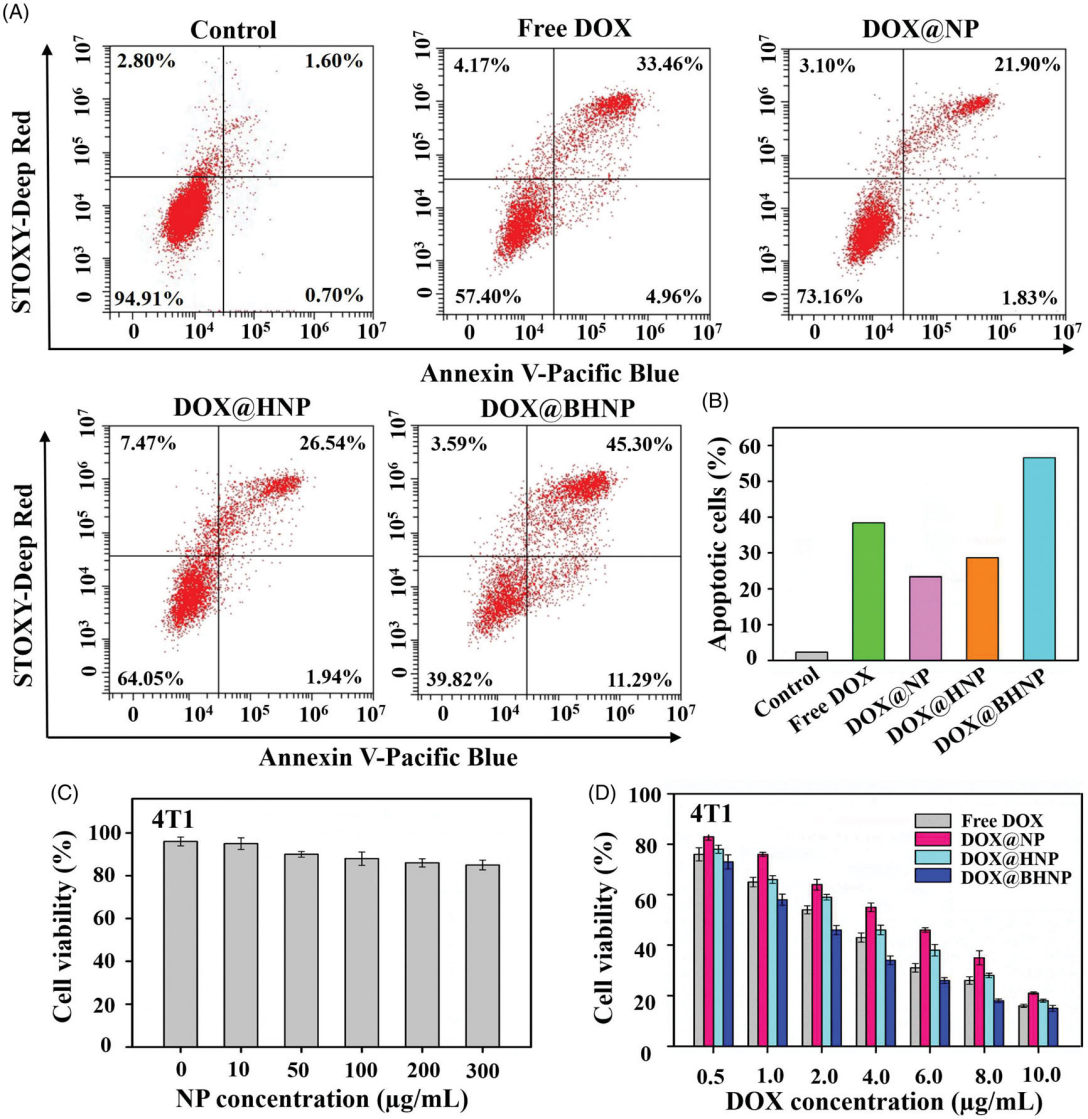
(A) Representative images of apoptosis studied by flow cytometry. The 4T1 cells were co-cultured with different reagents (free DOX, DOX@NP, DOX@HNP, and DOX@BHNP at a DOX concentrations of 6 µg mL^−1^) for 24 h and (B) quantitative analysis of apoptosis. (C) Cell viability of 4T1 cells after treatment with different concentrations of NP for 48 h. (D) Cell viability of 4T1 cells after treatment with different reagents (free DOX, DOX@NP, DOX@HNP, and DOX@BHNP) at different DOX concentrations for 48 h. The cells without treatment were used as control. Error bars indicate S.D. (*n* = 3).

**Figure 4.**
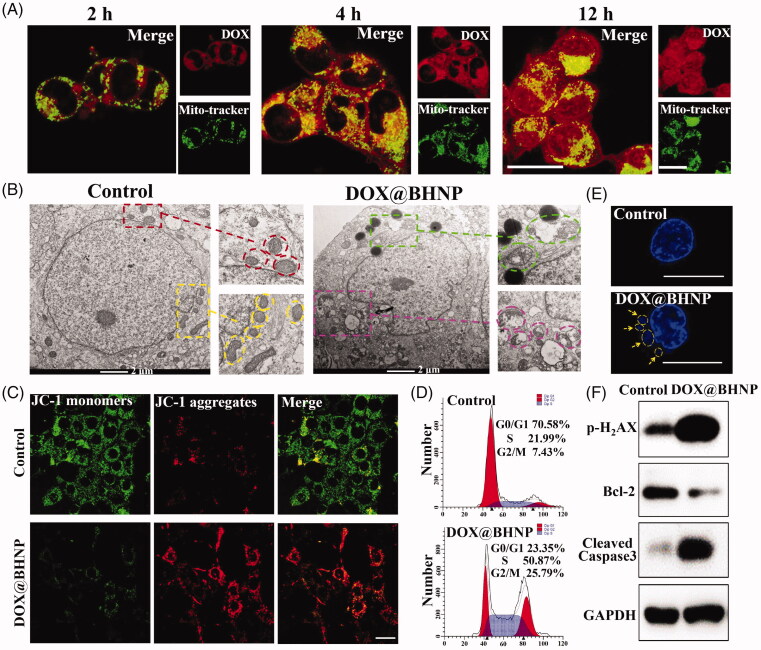
(A) CLSM images of the distribution of DOX in the organelles of 4T1 cells after incubation with DOX@BHNP for 2, 4, and 12 h (scale bar: 30 µm). (B) Mitochondrial morphology of 4T1 cell after incubation with DOX@BHNP for 24 h (scale bar: 2 µm). (C) Mitochondrial membrane potential of 4T1 cells after incubation with DOX@BHNP for 24 h (scale bar: 30 µm). (D) Flow cytometry analysis of cell cycle in 4T1 cells after incubation with DOX@BHNP for 24 h, and the specific cell cycle phases are indicated: G0/G1 = gap 1; S = synthesis; G2/M = gap 2 and mitosis (DOX concentrations: 6 µg mL^−1^). (E) Nuclear DNA leakage of 4T1 cells after incubation with DOX@BHNP for 24 h (scale bar: 10 µm). (F) Western blot assay of p-H_2_AX, Bcl-2, and cleaved caspase-3 expression in 4T1 cells after incubation with DOX@BHNP for 24 h. The cells without treatment were used as a control.

The acid-responsive cumulative drug release characteristics of DOX@BHNP were evaluated. Tris–HCl solutions of pH 7.4, 6.5, and 5.3 were used to simulate blood circulation, the acidic environment of the tumor microenvironment, and lysosomes, respectively. As shown in Figure 1(E), in a solution of pH 5.3, more than 80%±2.3 of DOX was released within 10 h, revealing that DOX@BHNP could release DOX efficiently under the acidic conditions in the tumor lysosome. In contrast, the DOX released from DOX@BHNP nanoparticles dramatically decreased to 45%±2.4 and 25%±3.7 at pH 6.5 and pH 7.4, respectively. The pH-sensitive release mechanism of DOX from DOX@BHNP is mainly because the CaCO_3_ component in the nanoparticle skeleton is easily decomposed under acidic conditions, resulting in the control of DOX release. Given the slightly acidic environment (pH 5.0–6.0) of cancer endosomes and the weakly alkaline environment (pH 7.4) in the blood and normal cells, this feature is suitable as an acid trigger for improving the rate of intracellular drug release and reducing the incidence of side effects (Gao et al., 2014; Dizaj et al., 2015; Liu et al., 2020).

### 3.2. Cellular uptake

After 4 h of incubation with different reagents, the cellular uptake behavior of DOX by 4T1 tumor cells was investigated via CLSM. The cells nuclei were labeled with Hoechst 33342 (blue fluorescence), and red fluorescence was observed from DOX. As shown in Figure 2(A), the accumulation of red fluorescence in the cytoplasmic region of the cells in the free DOX group was better than that in the DOX@NP group, likely because small DOX molecules can enter the cells through the rapid passive diffusion, which is better than the unfunctionalized carrier that enters the cell through endocytosis as verified in previous studies (Zhang et al., 2020; Liu et al., 2021). In contrast, the cells treated with HA- or BHA-modified nanoparticles showed significantly enhanced red fluorescence compared to the free DOX group. As expected, the highest fluorescence intensity was detected in the 4T1 cells treated with dual-functionalized nanoparticles (DOX@BHNP), showing the synergetic tumor-targeting effects of biotin and HA, resulting in an increase in intracellular DOX concentration. The intracellular DOX concentrations in the cells treated with different reagents were quantitatively studied via flow cytometry (Figure 2(B)), and the MFI of cells incubated with DOX@BHNP was significantly higher than that of the cells treated with free DOX, DOX@NP, or DOX@HNP. Treatment of HeLa and MCF-7 cells exhibited similar results and is presented in Figure S5–S6.

**Figure 5.**
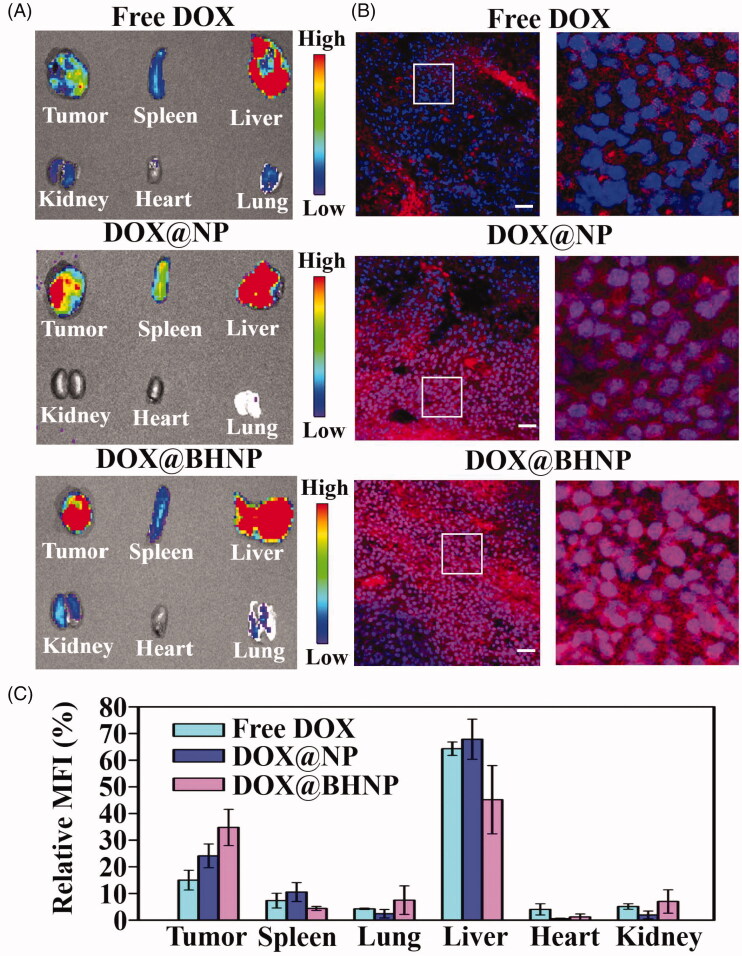
(A) Overlaid fluorescence images of the tumors and main organs of the 4T1 tumor-bearing mice at 24 h post injection of free DOX, DOX@NP, and DOX@BHNP, respectively. (B) DOX distribution in sections of tumor tissues as determined via CLSM. Scale bar: 30 µm. (C) Relative mean fluorescence intensity (MFI) of the major organs and tumors of free DOX, DOX@NP, and DOX@BHNP nanoparticles treated mice at 24 h post injection.

**Figure 6.**
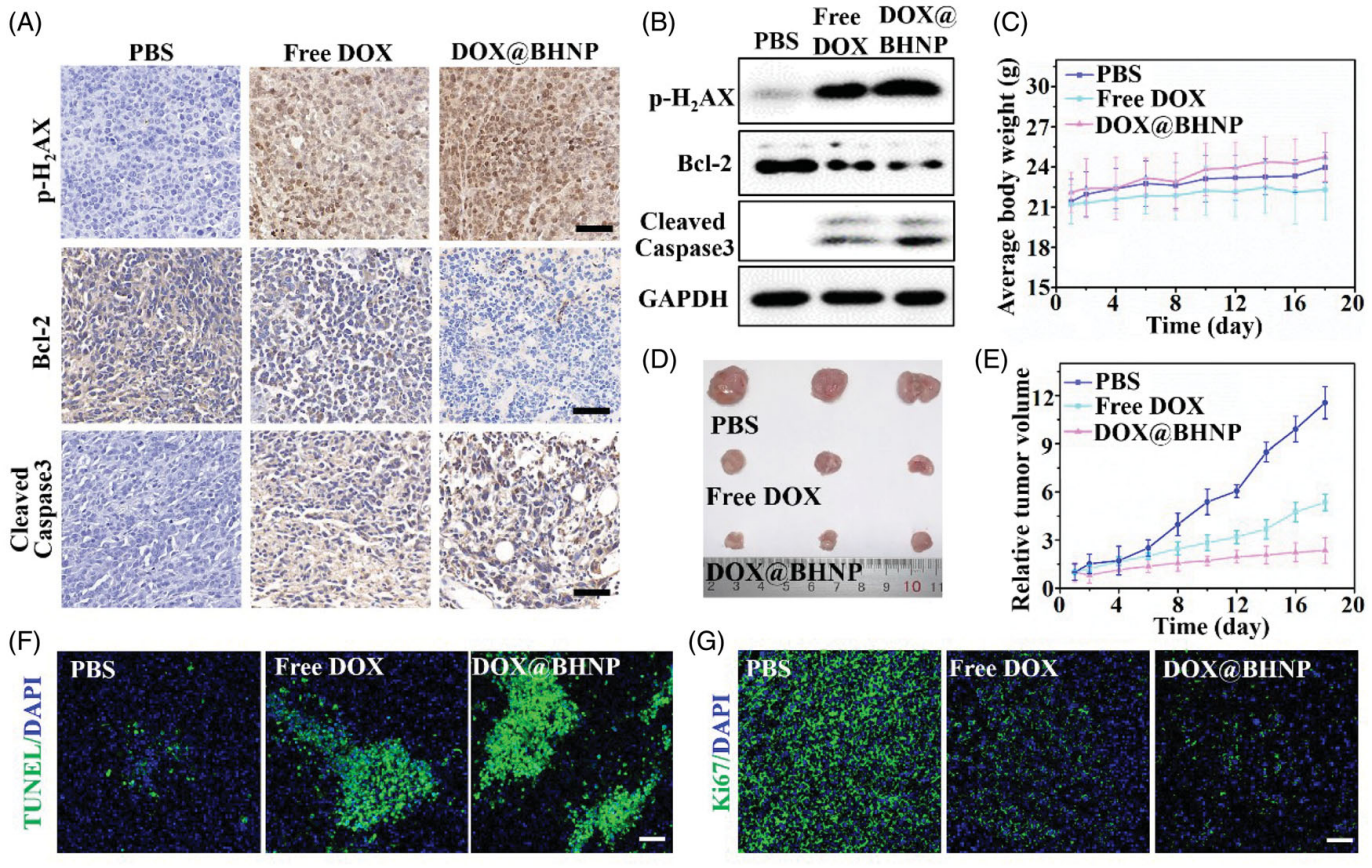
(A) Immunohistochemistry and (B) western blot assay of p-H_2_AX, Bcl-2, and cleaved caspase-3 expression in the tumor tissues. (C) Average body weight of the mice in the different treatment groups during the experimental period. (D) Photographs of tumors isolated from the different groups on the 18th day after different intravenous administration of the treatments. (E) Relative tumor volume in the mice after the intravenous administration of different treatments during the experimental period. (F, G) Representative histological analysis images of TUNEL and Ki67-stained tumor sections obtained on the 18th day after treatment. The mice were treated with PBS, free DOX, and DOX@BHNP. Scale bar: 50 µm. Data are reported as mean ± S.D.

### 3.3. Cytotoxicity evaluation

The cytotoxicity of our hybrid nanoparticles was evaluated using the MTT assay in 4T1, HeLa, and MCF-7 cells after treatment with different reagents. As depicted in Figure 3(C), a concentration ranges of the blank solution (NP) from 0 to 300 µg mL^−1^ did not show obvious cytotoxicity (cell viability >90%), which was attributed to all components in our drug delivery system having good biocompatibility within the given dose range. The antitumor effects of the drug-loaded hybrid nanoparticles were also studied and are shown in Figure 3(D) and Figure S7. As expected, after BHA functionalization, DOX@BHNPs with active targeting ability could be easily absorbed by tumor cells and showed the highest tumor cell inhibitory activity. For example, the median lethal dose of DOX@BHNP to 4T1 cells is approximately 1–2 µg mL^−1^, which is about half that of the free DOX group (2–4 µg mL^−1^). Flow cytometry analysis of Annexin V-Pacific Blue/SYTOX double staining showed that the number of apoptotic 4T1 cells was significantly increased when cells were treated with DOX@BHNP (Figure 3(A,B)), which was consistent with the results of the MTT assay and CLSM visualization.

DOX has been reported to kill tumor cells by inducing nuclear DNA damage or mitochondrial depolarization. Herein, the distribution of DOX in subcellular organelles delivered using a multifunctional carrier was also studied. As shown in Figure 4(A), DOX mainly accumulated in the nuclear region and the mitochondria of the cells after co-incubation with DOX@BHNP for 12 h, which further indicated that DOX could be released from the BHNP platform in the endo/lysosome in an acidic environment and diffused to the nuclei and mitochondria. After the 4T1 cells were treated with DOX@BHNP for 24 h, their morphology and mitochondrial membrane potential were observed via bio-TEM and fluorescence spectrophotometry, respectively. As shown in Figure 4(B), the mitochondrial morphology showed obvious destruction from the release of DOX compared to the untreated cell. Additionally, compared with normal mitochondria (showing red fluorescence), cells incubated with DOX@BHNP exhibited more green fluorescence, indicating that the mitochondria were damaged (Figure 4(C)). A comparison of the incidence of apoptosis between untreated and DOX@BHNP-treated 4T1 cells showed that the cells were arrested in the G2/M phase in DOX@BHNP-treated cells (Figure 4(D)), indicating a delay in the cell cycle progression. As shown in Figure 4(E), the nucleus of 4T1 cell in the DOX@BHNP treatment group is no longer a regular circle, and there is obvious DNA leakage around it. At the molecular level, the up-regulation of phosphorylation of p-H_2_AX (DNA damage related protein), cleaved caspase-3 (pro-apoptosis protein), and down-regulation of Bcl-2 (anti-apoptosis protein) are clearly observed in the 4T1 cells following DOX@BHNP treatment (Figure 4(F)). These results demonstrate that the targeting drug delivery systems can damage mitochondria, induce nuclear DNA leakage, and inhibit the proliferation of tumor cells.

### 3.4. *In vivo* therapeutic evaluation

Before *in vivo* application, the biocompatibility of DOX@BHNP nanoparticles was evaluated by hemocompatibility assays. As presented in Figure S8A,B, in all nanoparticles concentrations ranging from 100 to 600 μg mL^−1^, hemolysis was less than 6%. Moreover, the morphology of erythrocytes did not change under this series of nanoparticle concentrations, while the erythrocytes were lysed in the positive control (H_2_O solution) (Figure S8C). This indicates that the drug delivery system has good blood compatibility and can be used *in vivo*. Then, the tumor biodistribution of DOX@BHNP was monitored using noninvasive near-infrared optical imaging. Briefly, free DOX, DOX@NP and DOX@BHNP were intravenously injected into 4T1 tumor-bearing mice, after administration for 24 h, the mice were sacrificed, and their major organs and tumor tissues were collected. The fluorescence signal of DOX in the different tissues and organs was imaged using a small animal imaging system. As shown in Figure 5(A), due to the enhanced permeability and the retention (EPR) effect of nanoparticles, tumor in DOX@NP-treated mice shows a stronger fluorescence signal than mice administered with free DOX. In addition, owing to the active targeting property of the DOX@BHNP, the fluorescence signal in the DOX@BHNP treated tumor tissue is the strongest, suggesting that the BHA functionalized vector can effectively accumulate DOX in tumors. Figure 5(C) shows the relative fluorescence signal of DOX in tumors and main organs. It is worth noting that the mice in the DOX@NP and DOX@BHNP groups showed little DOX accumulation in the heart compared to those treated with free DOX, suggesting that the biocompatibility of DOX-loaded CaCO_3_ nanoparticles can effectively reduce the cardiotoxicity caused by free DOX (Dwivedi et al., 2020). Further, the distribution of DOX in the sections of the tumor tissues slides was determined via CLSM (Figure 5(B)). Compared with the tumor sections treated with free DOX and DOX@NP, those in the DOX@BHNP group showed a more extensive distribution of red fluorescence throughout the entire tumor tissue section. This result indicated that the dual-functionalized drug delivery system could deliver DOX much deeper into the tumor tissue than free DOX or no targeted system.

*In vivo* antitumor therapeutic evaluations were performed by intravenously injecting DOX@BHNP into 4T1 tumor-bearing mice when their tumor volumes have already reached ∼80 mm^3^. After determining the tumor targeting ability of DOX@BHNP during antitumor therapy, it was observed that administration of DOX@BHNP had no significant effect on the body weight of the mice (Figure 6(C)) while retaining its therapeutic efficacy. As shown in Figure 6(E), the tumor size in mice treated with free DOX or PBS increased up to 5 or 12 times after five consecutive injections. On the contrary, with its excellent tumor targeting and acid-triggered drug release abilities, DOX@BHNP significantly suppressed tumor growth, showing only a twofold increase in tumor size at day 18 (from ∼83 to ∼155 mm^3^). The morphology of tumors isolated from the different groups at the end of the study is shown in Figure 6(D). In particular, the results of the histological analysis further confirmed that DOX-loaded BHNPs were the most effective in inducing cell apoptosis (Figure 6(F)) and reducing cell proliferation (Figure 6(G)) among the treatments administered. As shown in Figure S9, H&E assays were used to analyze the histology of the major organs and tumors in each group after 18 days of first administration. A slight toxicity was observed in the heart tissue of the free DOX-treated group, which may be related to the non-targeted distribution of DOX. In contrast, the DOX@BHNP-treated mice showed no detectable pathological abnormalities or tissue damage in the liver, spleen, lung, heart, and kidney, when compared with the PBS-treated group. The IHC assay results revealed that in DOX@BHNP-treated mice, the positive expression of p-H_2_AX and cleaved caspase-3 was the strongest, and the expression of Bcl-2 was relative low in DOX@BHNP-treated mice (Figure 6(A)). Western blot assay shows that the expression of Bcl-2 is significantly suppressed after DOX@BHNP treatment and the expression of p-H_2_AX and cleaved caspase-3 is up-regulated (Figure 6(B)). The results of western blot assay are good consistent with IHC assay, indicating that DOX@BHNP could successfully induce mitochondrial damage, nuclear DNA leakage, and tumor cell apoptosis *in vivo*. Overall, the *in vivo* antitumor results were consistent with the *in vitro* cytotoxicity data and clearly indicated that the dual-functionalized drug-loaded nanoparticles had significant anticancer effects and low toxicity, and could greatly improve the efficacy of cancer therapy in the future.

## 4. Conclusions

In summary, a BHA-guided dual-functionalized CaCO_3_-based drug delivery system (DOX@BHNP) with target specificity and acid-triggered drug-releasing capability was developed to effectively deliver DOX to the mitochondria and nuclei of tumor cells. The drug-loaded nanoparticles have a well-defined spherical structure and are homogeneously dispersed as individual particles, as observed via TEM. Their average diameter was approximately 209 ± 10 nm, and no significant dimensional changes were observed via DLS after being placed in dark for six days, showing that the nanoparticles had good stability. The batch-to-batch variation of DOX loading level in DOX@BHNP system was negligible under eight independent experiments. The *in vitro* antitumor experiments demonstrated that DOX@BHNP system has remarkable tumor accumulation, good pH sensitivity, significant mitochondrial destruction and nuclear DNA damage. Virtually, an effective solid tumor inhibition was observed *in vivo*, suggesting that the hybrid nanosystem could be used as an effective and safe carrier for the delivery of chemotherapeutic drugs for use in cancer treatment.

## Supplementary Material

Supplemental Material
