# Supplementary material for: Targeted delivery and stimulus-responsive release of anticancer drugs for efficient chemotherapy
Source: Drug Deliv. 2021 Oct 20;28(1):2218–28. doi: 10.1080/10717544.2021.1986602 (PMC8530493; doi:10.1080/10717544.2021.1986602)
Supplement: Supplemental Material [file IDRD_A_1986602_SM9725.doc]

Supporting Information

**Targeted delivery and stimulus-responsive release of anticancer drugs for efficient chemotherapy**

*Lei Qiao^c^, Xue Yuan^a^, Hui Peng^a^, Guisong Shan^a^, Min Gao^e^, Xiaoqing Yi ^d*^, Xiaoyan He ^a,b**^*

^a^ School of Life Sciences, Anhui Medical University, Hefei 230032, China

^b^ Inflammation and Immune Mediated Diseases Laboratory of Anhui Province, Anhui Medical University, Hefei 230032, China

^c^ School of Basic Medical Sciences, Anhui Medical University, Hefei 230032, China

^d^ Key Laboratory of Prevention and Treatment of Cardiovascular and Cerebrovascular Diseases, Ministry of Education, Gannan Medical University, Ganzhou 341000, China.

^e^ Department of Respiratory and Critical Care Medicine, The First Affiliated Hospital of Anhui Medical University, Hefei 230022, China

^*^ Corresponding author.

^* *^ Corresponding author.

Email address: keyi0115@126.com (X. Yi); hexiaoyan@ahmu.edu.cn (X. He).

**MATERIALS AND METHODS**

**Materials**

HeLa, MCF-7, and 4T1 cells were obtained from the China Center for Typical Culture Collection (Wuhan, China) and were cultured in DMEM or RPMI-1640 (Gibco) supplemented with 10% fetal bovine serum (FBS) and 2 mg mL^-1^ penicillin-streptomycin at 37 °C in a humidified 5% CO_2_ atmosphere.

**Animals**

All animal procedures were performed in compliance with the relevant laws and guidelines of the Laboratory Animal Center of Anhui Medical University (approval number: LLSC 20190715). Briefly, healthy female BALB/c mice (5-6 weeks old) were obtained from the Anhui Medical University Laboratory Animal Center, and 1 × 10^6^ 4T1 cells suspended in 100 μL PBS were subcutaneously injected into the right hind leg of the mice to establish tumor models. Tumor growth was measured using a caliper, and tumor volume was calculated using the formula: volume = (tumor length × tumor width^2^)/2. Relative tumor volume was defined as *V_d_*/*V_0_*, where *V_d_* and *V_0_* are the tumor volumes on the day of measurement and on the first day of treatment, respectively.

**Synthesis and characterization of biotinylated hyaluronic acid (BHA)**

10 mL of DMF was used to dissolve 10 mmol of biotin, then 12 mmol of DCC and 12 mmol of NHS were added under stirring at 25 ℃ and reacted for 2 h. Then, the solvent was evaporated, and the precipitate was dissolved in acetone to obtain biotin–NHS. Subsequently, biotin–NHS was dissolved in DMF, and 5 mL of ethylenediamine trimethylamine was added and stirred for another 24 h. After the reaction, the solvent was removed using a rotary evaporator, ether was added at a low temperature (4 °C) to completely precipitate the product, and the resulting precipitate was dried in vacuum to obtain biotin-ethylenediamine. Additionally, 170 µg of HA was dispersed in 1 mL PBS (pH 6.0) and activated with a catalyst EDC/HoBt (-COOH: EDC: HoBt =1:1.2:1.2 molar ratio) at room temperature for 1 h, and then 70 µg of biotin-ethylenediamine was added and continuously stirred at room temperature for another 24 h. The product was dialyzed for three days in deionized water using a dialysis bag (MWCO 3500) to remove the unreacted reagents and then lyophilized to obtain biotinylated hyaluronic acid (BHA). Finally, the product was analyzed using ^1^H NMR spectroscopy (Bruker AM 400).

**Drug loading capacity (DLC) and drug encapsulation efficiency (DEE) determination**

Briefly, the DLC (%) and DEE (%) values were calculated using the formula: DLC (%) = [(*W_m_* - *W_e_*)/*W_NP_*] × 100% and DEE (%) = [(*W_m_* - *W_e_*)/*W_m_*] × 100%. Where *W_m_* is the total weight of the drug added, *W_e_* is the weight of the drug that was not encapsulated, and *W_NP_* is the total weight of the drug-loaded micelles.

***In vitro* cytotoxicity assay**

Cells suspended in 100 µL of culture medium containing 10% FBS were seeded into a 96 well plate (5 × 10^3^ cells per well) and incubated at 37 °C and 5% CO_2_ for 24 h. After incubation for 24 h, the spent medium was removed and the cells were treated with culture medium containing free DOX and/or DOX-loaded nanoparticles at various concentrations for another 48 h. After 48 h, 20 µL of MTT solution in PBS (5 mg mL^-1^) was added and the cells were incubated at 37 °C for 4 h. Finally, the supernatant was carefully removed and 100 µL of DMSO was added to dissolve the formazan crystals produced by the viable cells. When the mixture became homogenous, a microplate reader (Bio-Rad 550) was used to determine the OD value at 570 nm. Cell viability was calculated as follows: Cell viability = OD_treated_/OD_control_ × 100%. Where OD_treated_ was the OD of the cells treated with a particular agent and OD_control_ was the OD of the untreated cells grown in 100 µL culture medium containing 10% FBS.

**Mitochondrial membrane potential assay**

The 4T1 cells were suspended in 1 mL culture medium containing 10% FBS and seeded into a 35-mm glass-bottom culture dish (MatTek) (1 × 10^4^ cells per well) and incubated at 37 °C for 24 h. The culture medium was then replaced with 1 mL of fresh medium containing DOX@BHNP (at a DOX concentration of 6 μg mL^-l^). After co-incubation for 24 h, the cells were carefully washed with 1 mL of PBS for three times, and then stained with 5,5’,6,6’-tetrachloro-1,1’,3,3’-tetraethylbenzimidazolylcarbocyanine iodide (JC-1) and observed via confocal laser scanning microscopy (CLSM) (Zeiss, LSM800). When JC-1 binds to normal mitochondria (with high mitochondrial membrane potential), it exhibits red fluorescence. In contrast, when JC-1 binds to damaged mitochondria (with low mitochondrial membrane potential), it exhibits green fluorescence.

**Western blot assay**

The 4T1 cells were seeded in a 6-well plate at a density of 2 × 10^5^ cells in 2 mL of medium per well and incubated at 37 °C for 24 h. Then the medium was replaced by 2 mL of fresh culture medium containing particular formulations with 6 μg mL^-1^ DOX, and then the cells were cultured at 37 °C for 48 h. The treated cells were rinsed twice with PBS, lysed and re-suspended in sodium dodecyl sulfate (SDS) sample buffer containing 1% β-mercaptoethanol. Total protein extracts were subjected to SDS-polyacrylamide gel electrophoresis (PAGE). After electrophoresis, the proteins were transferred to poly (vinylidene fluoride) (PVDF) membranes (Millipore). After treated with TBST (Tris-buffered saline with Tween-20) containing 5% milk for 1 h, the membranes were incubated with the primary antibody overnight at 4 °C. After washing, the PVDF membranes were incubated with the secondary antibody for 2 h at 25 °C. After that, the protein on PVDF membrane were visualized with Tanon 4600SF (China).

**Hemolysis test**

The release of hemoglobin from mice blood cells was used to evaluate the hemolytic activities of DOX@BHNP nanoparticles with different concentrations; 100 μL red blood cell (10%) suspension mixed with 900 μL H_2_O (served as positive control), 900 μL NaCl (0.99%, served as negative control), and different concentrations of DOX@BHNP, respectively. After being kept at 37 °C for 6 h in the dark, the dispersion states of the erythrocytes were observed by optical microscopic. After centrifugation, the hemolysis ratio of red blood cells was calculated using the following formula:

Hemolysis (%) = (*A_sample_* – *A_negative_*)/(*A_positive_ - A_negative_*) × 100%

Where *A_sample_, A_negative_,* and *A_positive_* refer to the absorption of material sample solution, negative control, and positive control at 570 nm, respectively.

**Biodistribution *in vivo***

After the tumor volume in 4T1 tumor-bearing mice reached approximately 300 mm^3^, Free DOX or DOX@BHNP (6 mg kg^−1^ of DOX) was intravenously injected *via* the tail vein. The mice were sacrificed, and the tumor tissues, livers, spleens, lungs, hearts, and kidneys were collected for imaging using a small animal imaging system (Ami HTX Spectrum, Spectral Instruments Imaging) after 24 h. In addition, the distribution of DOX in tumor tissue sections was also evaluated using CLSM.

**Immunohistochemical (IHC) assay**

Formalin-fixed paraffin-embedded tumor sections were used for IHC assay. Briefly, sections 5 μm thick from paraffin-embedded tumor sections were first heated to 60 °C for 1 h, deparaffinized with xylene (3 × 5 min) and washed with a graded series of ethanol. After using DAKO target repair solution to repair the antigen at 95-99 °C for 40 min, then the slides were washed with PBS and blocked with peroxidase blocking buffer (DAKO Company) for 5 min. Subsequently, the slides were incubated with relative antibody diluted in DAKO antibody solution for 1 h. The slides were incubated with peroxidaselabeled polymer for 30 min. After washing and staining with DAB + substrate-chromogen solution and hematoxylin, the slides were remounted and viewed under a MVX10 Macro View Dissecting scope equipped with an OlympusDP80 camera.

**
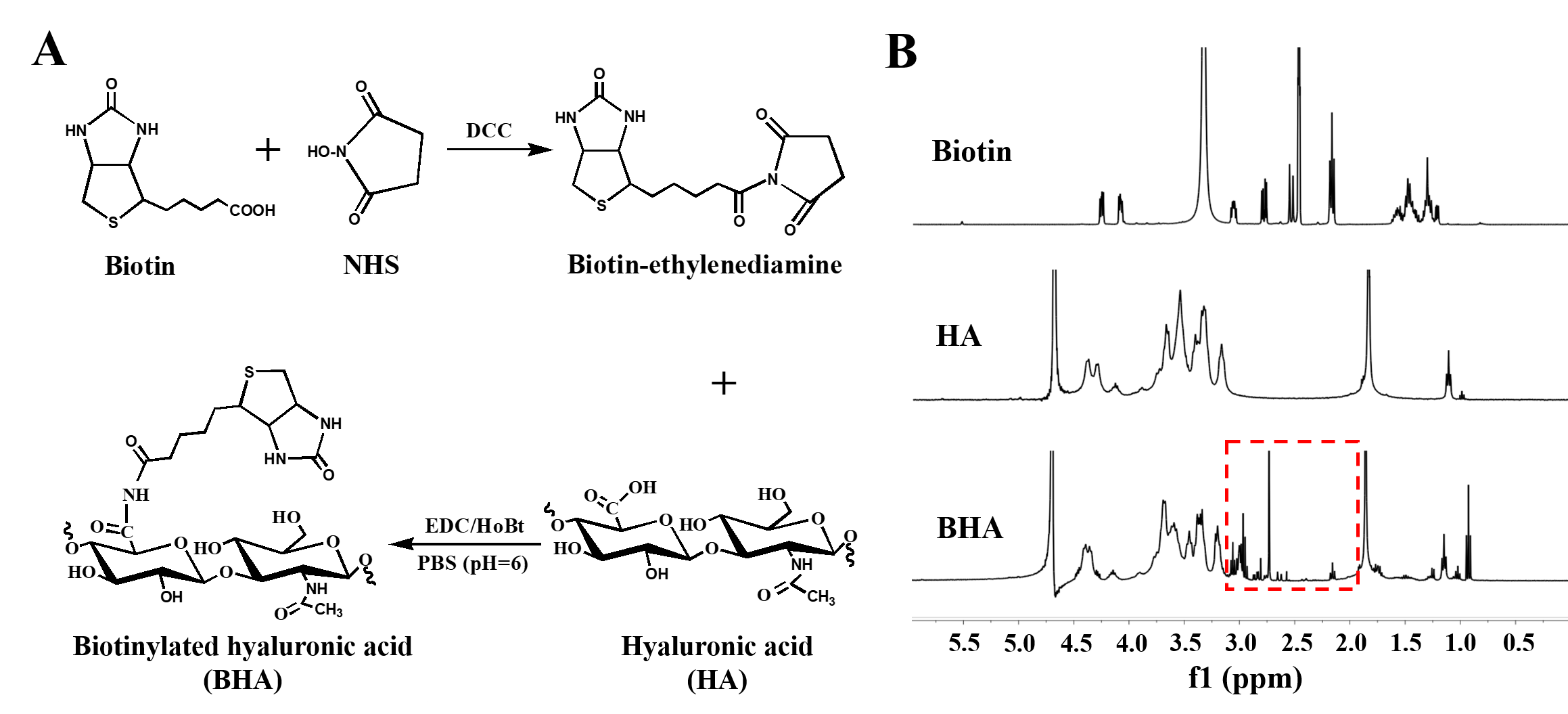
**

**Figure S1.** (A) Synthesis of biotin-conjugated hyaluronic acid (BHA). (B) ^1^H NMR spectra of biotin in DMSO-d6, HA, and BHA in D_2_O.


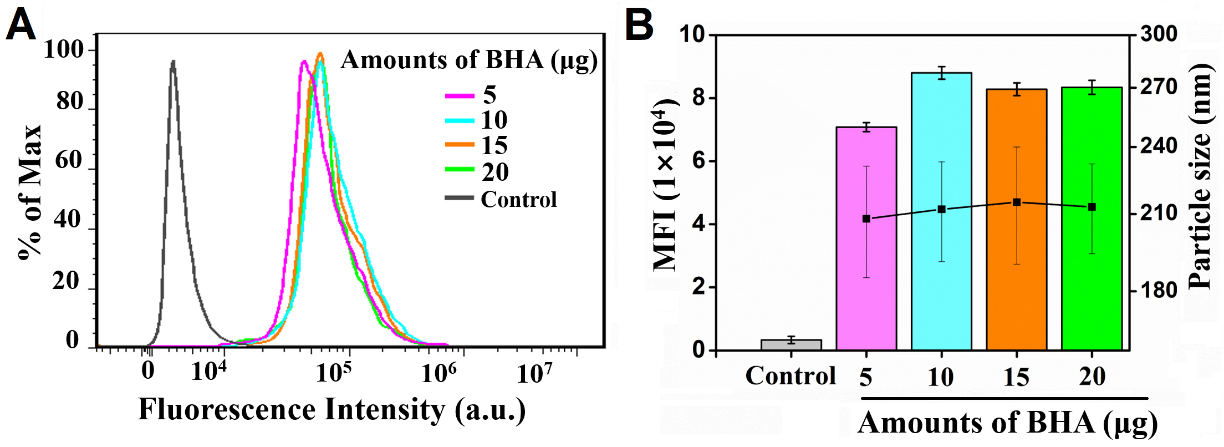


**Figure S2.** (A) Flow cytometry analysis of the intracellular DOX levels in 4T1 cells incubated with DOX@BHNP with different amounts of BHA for 4 h (DOX concentration: 6 µg mL^-1^). (B) The quantitative assessment of intracellular DOX in 4T1 cells incubated with DOX@BHNP with different amounts of BHA for 4 h (DOX concentration: 6 µg mL-1), and the particle size of DOX@BHNP with different amounts of BHA as analyzed via DLS. The cells without treatment as control. Scale bar: 30 µm. Error bars indicate s.d. (n = 3).


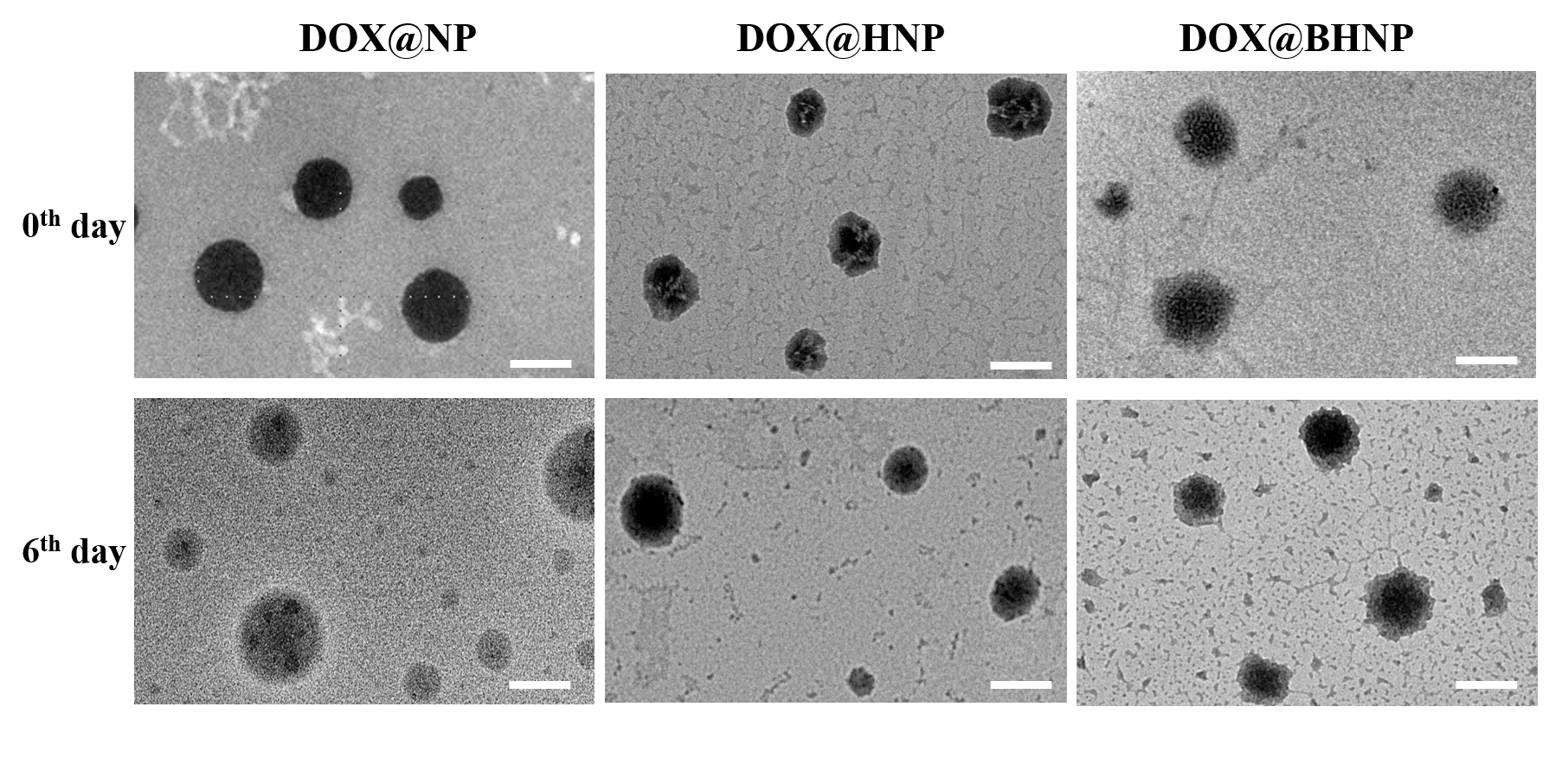


**Figure S3**. TEM images of DOX@NP, DOX@HNP and DOX@BHNP nanoparticles on the 0^th^ day and 6^th^ day, respectively. Scale bar: 200 nm.


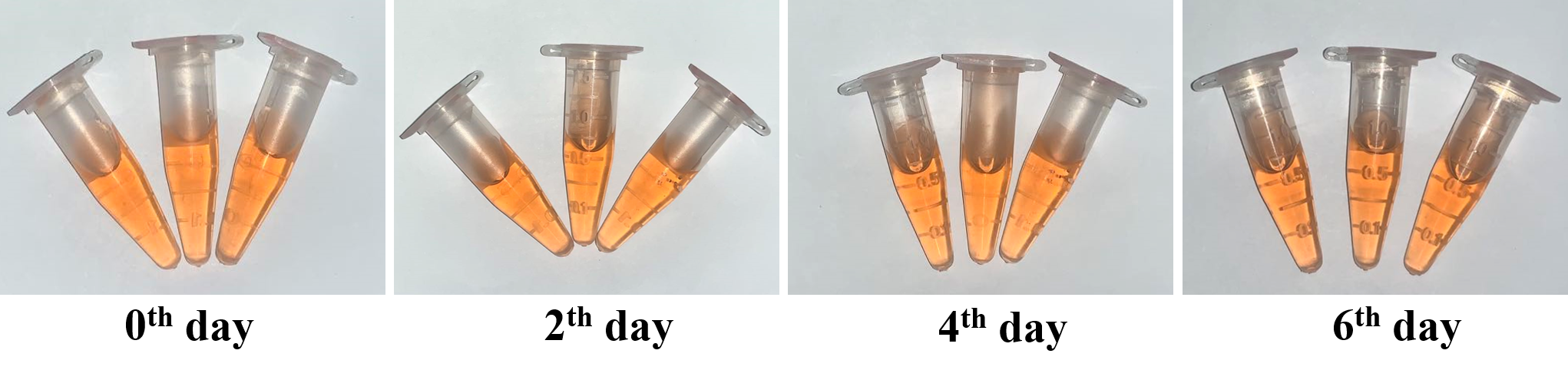


**Figure S4**. Photographs of DOX@BHNP nanoparticles solution on the 0^th^ day, 2^th^ day, 4^th^ day, and 6^th^ day, respectively.


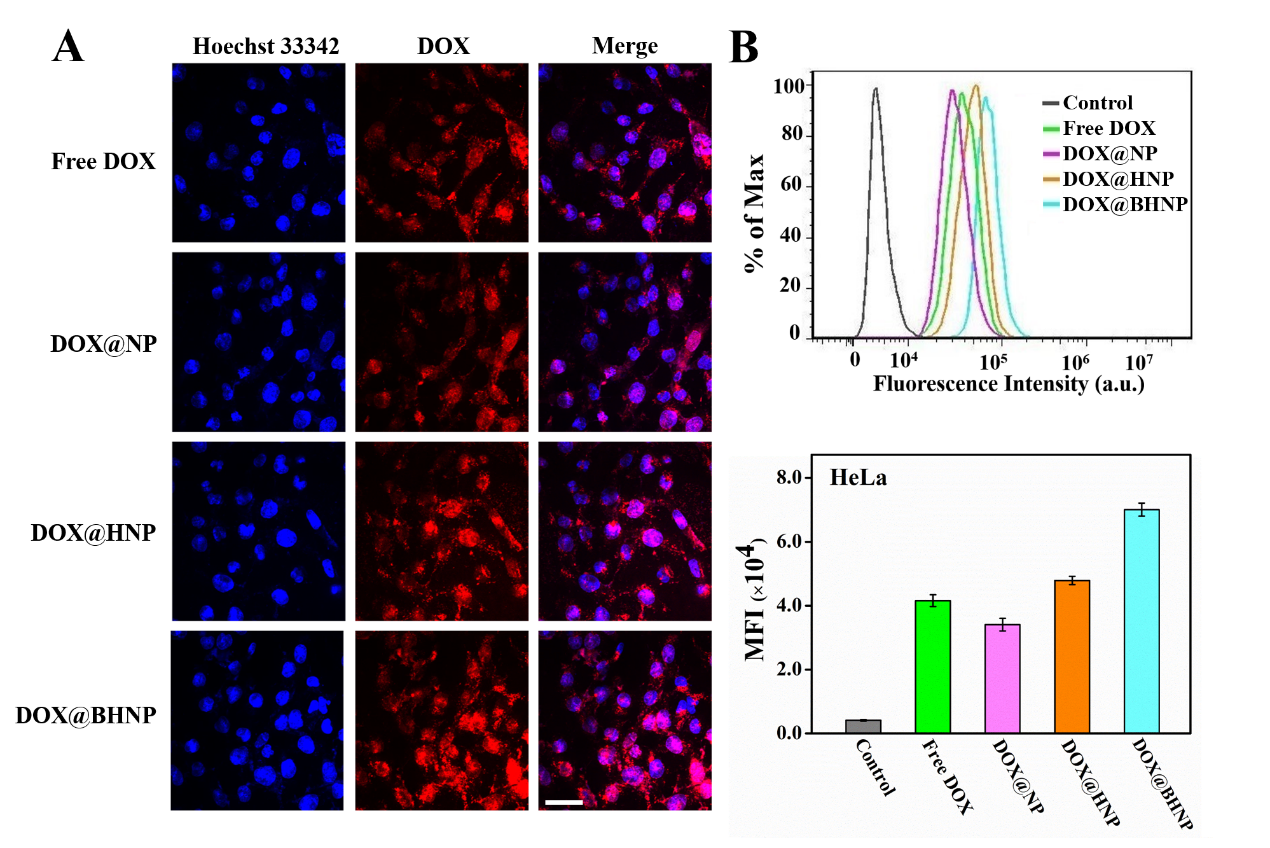


**Figure S5.** (A) CLSM images and (B) flow cytometry analysis of the intracellular uptake of DOX in HeLa cells incubated with different agents for 4 h (DOX concentrations: 6 µg mL^-1^, scale bar: 30 µm). Error bars indicate s.d. (n = 3).


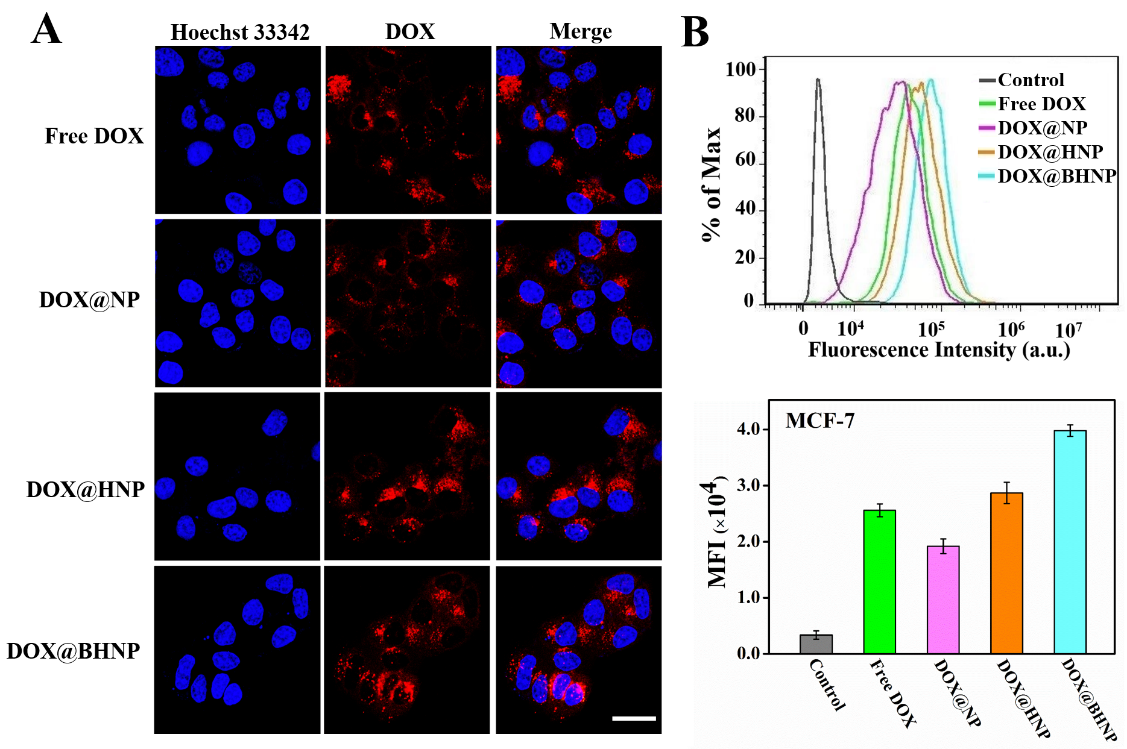


**Figure S6.** (A) CLSM images and (B) flow cytometry analysis of the intracellular uptake of DOX in MCF-7 cells incubated with different agents for 4 h (DOX concentrations: 6 µg mL^-1^, scale bar: 30 µm). Error bars indicate s.d. (n = 3).


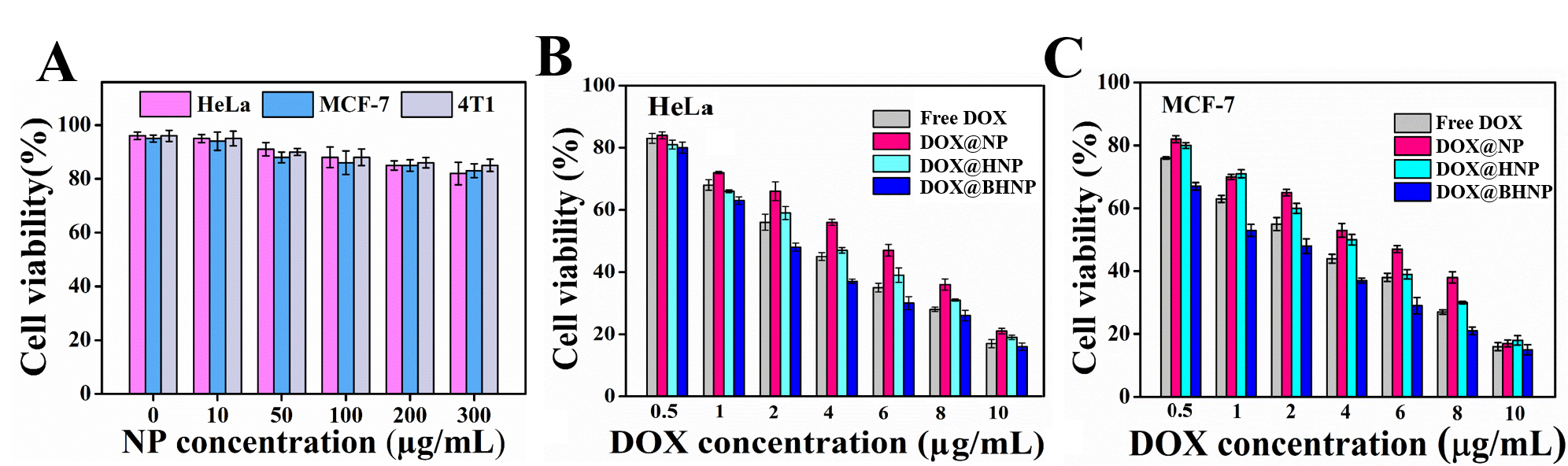


**Figure S7.** (A) Cell viability after treatment with different concentrations of NP for 48 h. (B-C) Viability of HeLa and MCF-7 cells after treatment with different reagents at different DOX concentrations for 48 h. Error bars indicate s.d. (n = 3).


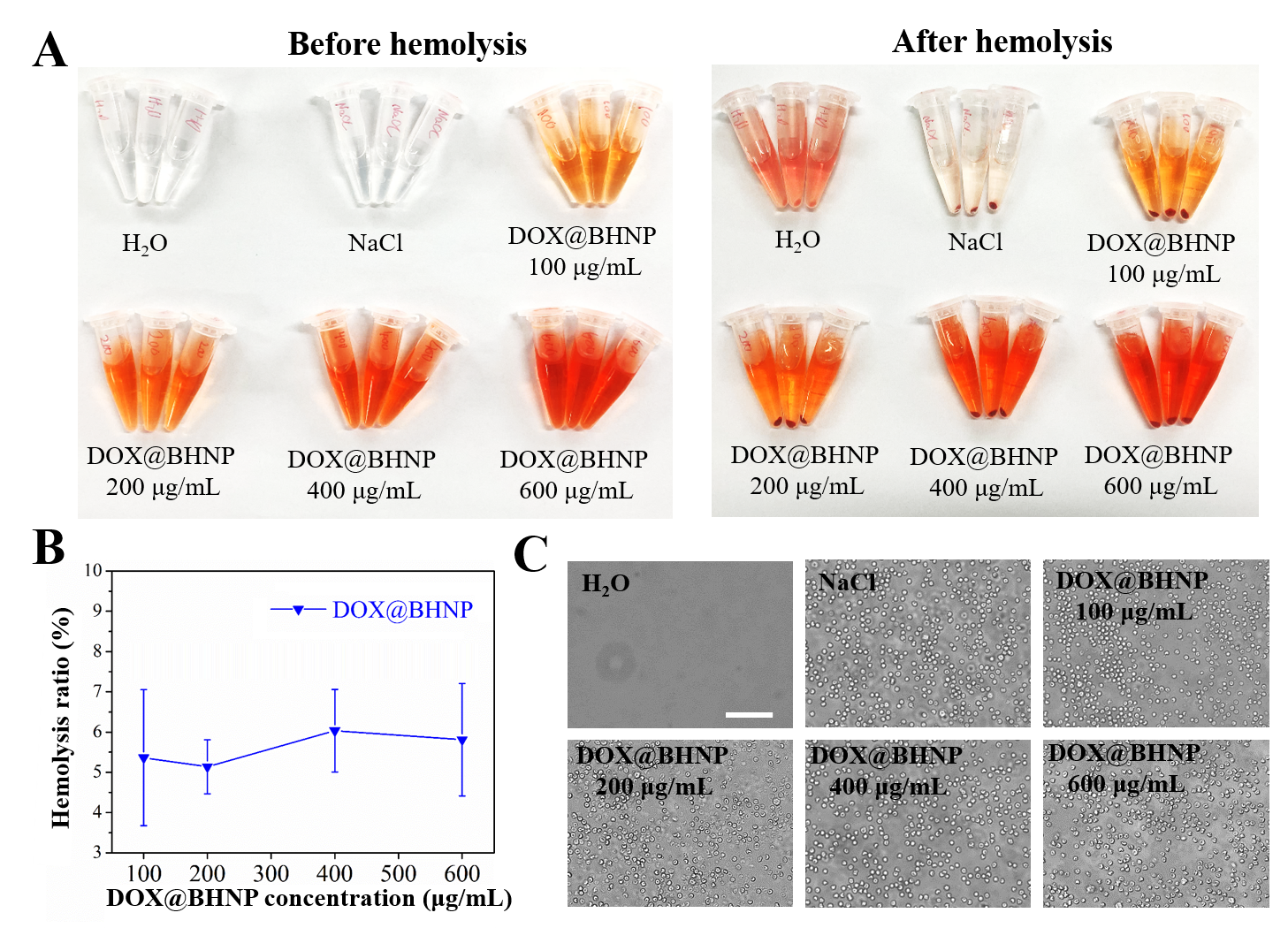


**Figure S8.** (A) Photographs of hemolysis assay after incubation with ultrapure water (positive control), 0.99% NaCl (negative control), and different concentrations of DOX@BHNP (100, 200, 400, and 600 mg mL^-1^, respectively) nanoparticles for 6 h in dark, respectively. (B) The hemolysis ratio induced by DOX@BHNP nanoparticles with different concentration incubated at 37 °C for 6 h in dark. (C) Optical microscopic observation of the dispersion states of the erythrocytes after incubated with different formulations for 6 h. Scale bar: 50 μm.


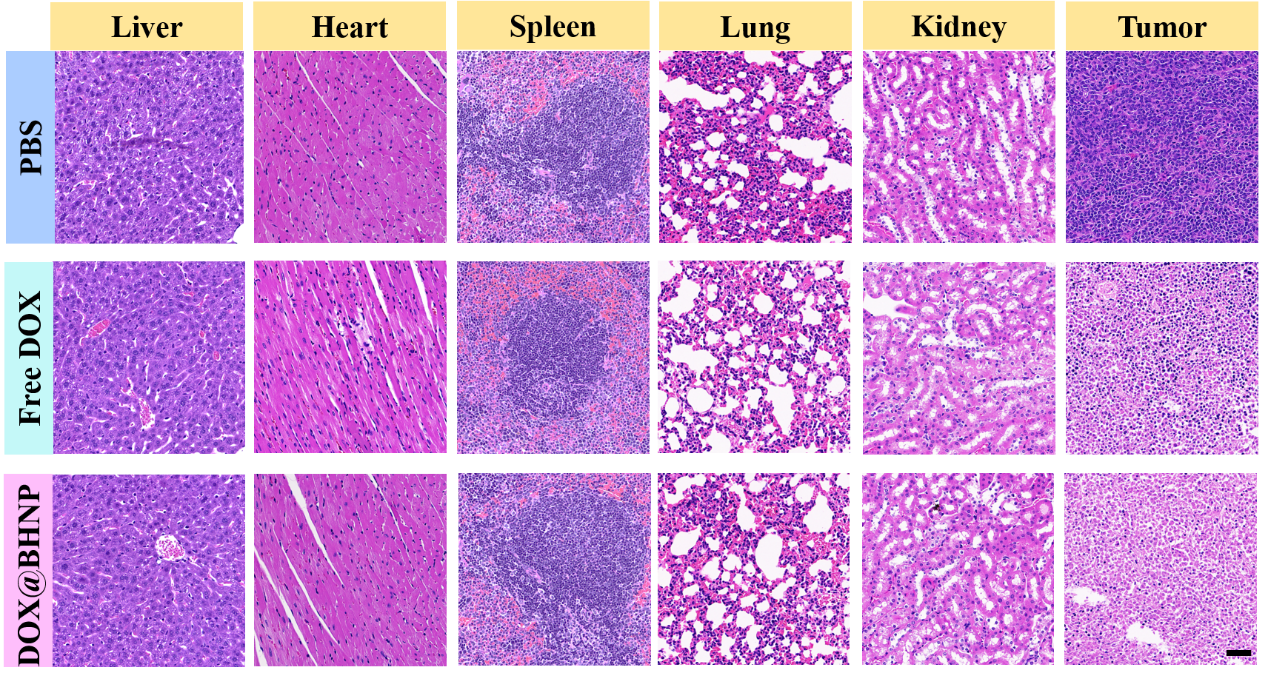


**Figure S9.** Representative histological sections of the main organs of the 4T1 tumor-bearing mice after intravenous injection with PBS, Free DOX, and DOX@BHNP, respectively. Scale bar: 50 µm.
